# Surface Passivation Method for the Super-repellence of Aqueous Macromolecular Condensates

**DOI:** 10.1021/acs.langmuir.3c01886

**Published:** 2023-10-05

**Authors:** Andrea Testa, Hendrik T. Spanke, Etienne Jambon-Puillet, Mohammad Yasir, Yanxia Feng, Andreas M. Küffner, Paolo Arosio, Eric R. Dufresne, Robert W. Style, Aleksander A. Rebane

**Affiliations:** † Department of Materials, ETH Zürich, 8093 Zürich, Switzerland; ‡ LadHyX, CNRS, Ecole Polytechnique, Institut Polytechnique de Paris, Palaiseau 91120, France; ¶ Department of Chemistry and Applied Biosciences, Institute for Chemical and Bioengineering, ETH Zürich, 8093 Zürich, Switzerland; § Life Molecules and Materials Laboratory, Programs in Chemistry and in Physics, New York University Abu Dhabi, P.O. Box 129188, Abu Dhabi, United Arab Emirates

## Abstract

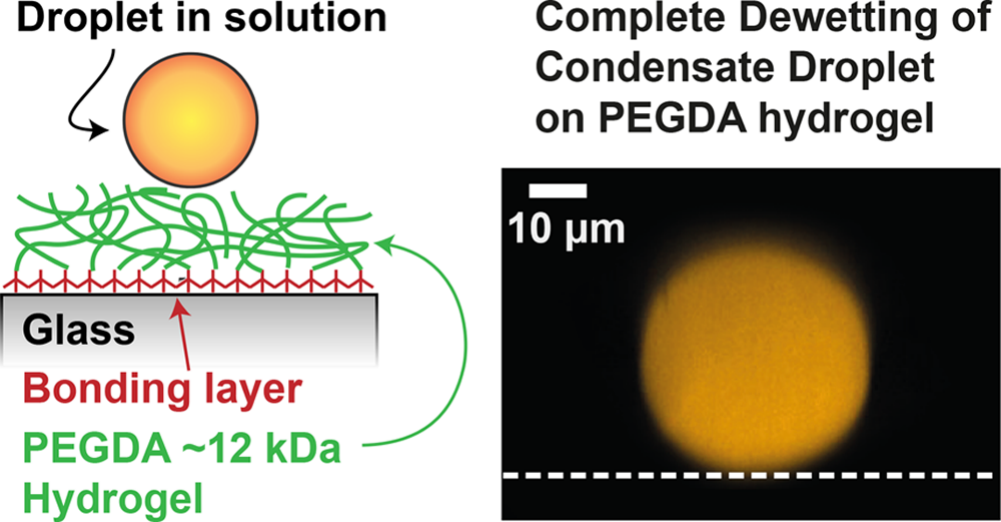

Solutions of macromolecules can undergo liquid–liquid phase separation to form droplets with ultralow surface tension. Droplets with such low surface tension wet and spread over common surfaces such as test tubes and microscope slides, complicating *in vitro* experiments. The development of a universal super-repellent surface for macromolecular droplets has remained elusive because their ultralow surface tension requires low surface energies. Furthermore, the nonwetting of droplets containing proteins poses additional challenges because the surface must remain inert to a wide range of chemistries presented by the various amino acid side chains at the droplet surface. Here, we present a method to coat microscope slides with a thin transparent hydrogel that exhibits complete dewetting (contact angles θ ≈ 180°) and minimal pinning of phase-separated droplets in aqueous solution. The hydrogel is based on a swollen matrix of chemically cross-linked polyethylene glycol diacrylate of molecular weight 12 kDa (PEGDA), and can be prepared with basic chemistry laboratory equipment. The PEGDA hydrogel is a powerful tool for *in vitro* studies of weak interactions, dynamics, and the internal organization of phase-separated droplets in aqueous solutions.

## Introduction

Aqueous solutions of macromolecules can undergo phase separation, forming droplets enriched with one or more components. This phenomena has garnered widespread interest in recent years thanks to the discovery that membraneless organelles inside cells appear to be phase-separated droplets of proteins and/or nucleic acids (biocondensates).^1,2^ Examples include P granules,^3^ P bodies,^4,5^ stress granules,^6^ and the nucleolus.^7^ The material properties and internal organization of biocondensates are thought to be important for biological function,^8,9^ and aberrations therein have been implicated in neurodegenerative diseases.^10,11^

A notable feature of biocondensates is their extremely low surface tension, with reported values of as low as γ ≈ 1 μN/m *in vivo*(3,12,13) and *in vitro*.^13−17^ Such low surface tension results in a strong tendency of biocondensates to adhere to most common substrates, including walls of test tubes and microscope slides (Figure S1),^18^ often to the detriment of *in vitro* experiments. For instance, droplet pinning causes underestimates of surface tension determined from the kinetics of fusion between two sessile droplets on a microscope slide.^13,16,19^ Similarly, wetting may introduce systematic errors into viscosity and viscoelasticity values obtained from passive and active microrheology.^15,17^ Further challenges arise when including macromolecular crowding in the buffer to more closely mimic the physicochemical properties of the cytoplasm.^20^ Crowding promotes nonspecific interactions that lead to even stronger adhesion of the biocondensates to microscope slides.^21^ Thus, accurate characterization of biocondensates *in vitro* requires surface passivation techniques that minimize interactions between the droplets and the experimental substrates.

Numerous surface passivation strategies have been developed to counteract droplet wetting.^22^ Microscope slides are typically coated with bovine serum albumin (BSA),^23,24^ fluorinated fluids,^13,25^ slippery omniphobic covalently attached liquids (SOCAL),^26^ or PEG-based polymer brushes^27−29^ or are treated to form a slippery liquid-infused porous surface (SLIPS).^30^ These treatments generally achieve selective dewetting conditions of either aqueous droplets (hydrophobic), nonpolar droplets (oleophobic), or certain combinations thereof (omniphobic). However, the creation of a surface coating that is super-repellent to biocondensates has remained elusive because they simultaneously present divergent chemistries on their surfaces, ranging from charged or polar to aliphatic or aromatic. Here, we put forward an optimized hydrogel surface that alleviates this issue by exhibiting complete dewetting of a broad range of phase-separated droplets in aqueous solution, thus allowing detailed quantitative characterizations of unperturbed droplets *in vitro*.

## Results and Discussion

### Experimental Design

We set out to formulate a substrate exhibiting complete dewetting of macromolecular droplets in aqueous solution (e.g. protein droplets). We focused on optically transparent coatings that could be applied to light microscopy glass slides for imaging and could be synthesized by nonexperts using basic chemistry laboratory equipment. Specifically, we chose to investigate the commonly used substrates of bare glass, a covalently attached polyethylene glycol brush on glass (PEG-silane), a slippery liquid-infused porous surface (SLIPS) of silicone oil, and a novel coating made of a chemically cross-linked polyethylene glycol diacrylate (PEGDA) hydrogel with a 12 kDa molecular weight.

Previously, we have successfully suppressed depletion-induced adhesion between a microscope glass and giant unilamellar vesicles by coating the glass with a low-molecular-weight PEGDA (700 Da) hydrogel.^31^ However, we found that this hydrogel still exhibited partial wetting of protein droplets, likely due to nonspecific attractions between PEGDA and protein^32^ (Figure S2). We postulated that by increasing the molecular weight of the PEGDA we could achieve a higher volume fraction of water and a lower volume fraction of polymer within the hydrogel,^33^ thereby making the hydrogel surface more similar to the solvent and reducing adhesive protein–hydrogel interactions. We therefore reformulated the hydrogel coating with 12 kDa PEGDA.

Detailed protocols for preparing these surfaces and synthesizing PEGDA are given in the Experimental Section. Briefly, we prepared PEG-silane glass slides by submerging glass coverslips in a toluene solution of 3-[methoxy(polyethyleneoxy)propyl]trimethoxysilane (PEG-silane) overnight.^15^ For SLIPS, we spray-coated glass coverslips with hydrophobic particles and then infused the resultant matrix with vinyl-terminated polydimethylsiloxane (silicone oil) by spin-coating.^34^ We prepared the PEGDA hydrogel by pretreating coverslips with the silane coupling agent 3-(trimethoxysilyl) propyl methacrylate.^31^ We then added a solution of 12 kDa PEGDA and photoinitiator (2-hydroxy-4-(2-hydroxyethoxy)-2-methylpropiophenone) to the pretreated glass slide, covered it with another glass slide coated with an antisticking film (RainX), and subsequently cured the hydrogel under UV light. The glass sandwich was then stored in an excess of Milli-Q water for up to several days. Prior to experiment, the sandwich was opened and the hydrogel equilibrated with the supernatant of the droplet suspension for 30 min. It should be noted that incompletely cured PEGDA mono- and oligomers might still be present in the hydrogel right after curing, which may result in time-dependent wetting behavior. It is therefore important to give ample time for the hydrogel to equilibrate with water and then the supernatant, which wash away any uncured PEGDA remaining in the gel. A schematic illustrating the key steps in PEGDA hydrogel preparation is shown in Figure S4.

### Static Contact Angle

**Figure 1:**
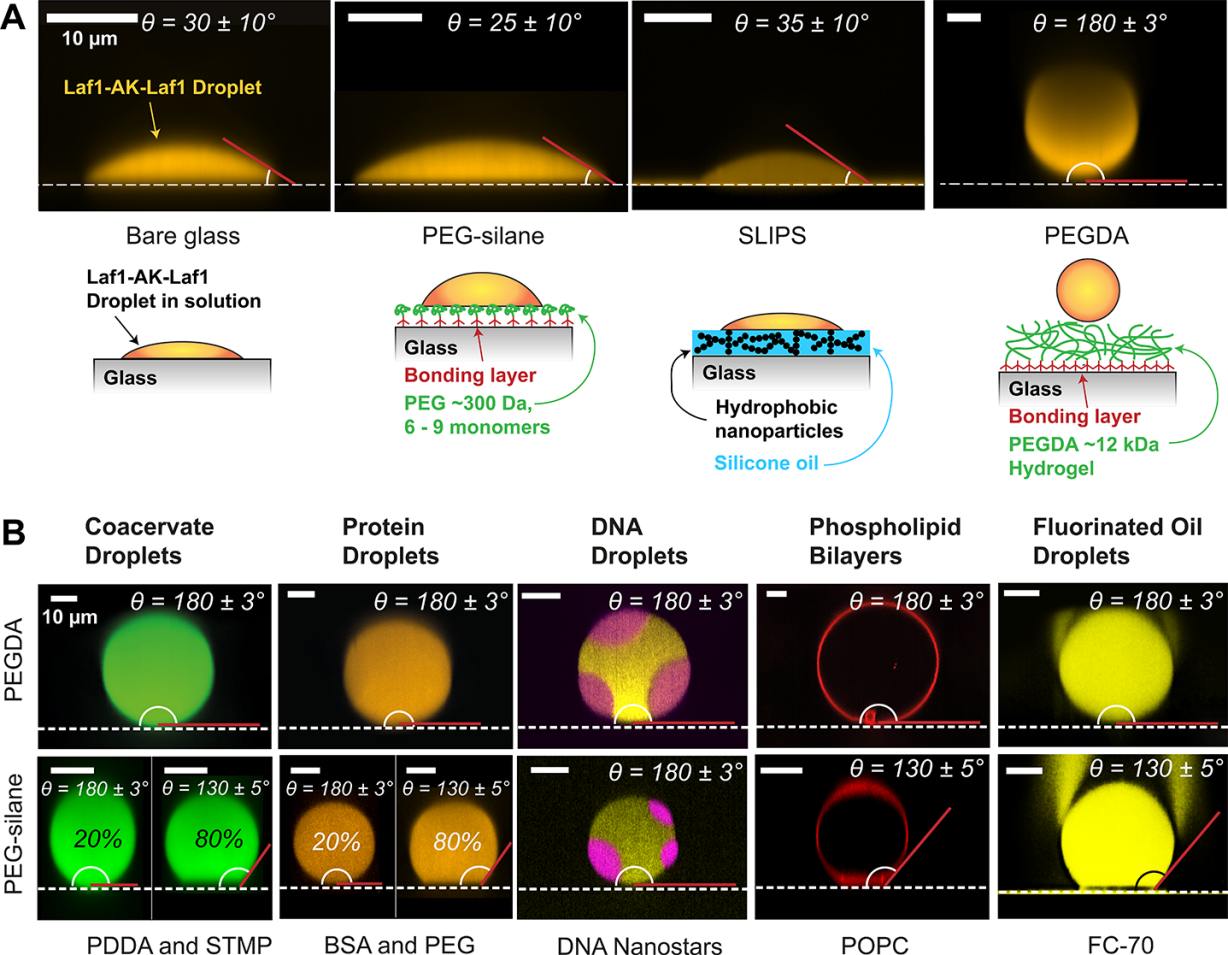
PEGDA hydrogel coating exhibits complete dewetting of droplets in aqueous solution. (A) Top: Side view and static contact angle of fluorescently labeled Laf1-AK-Laf1 droplets (orange) resting on different substrates (white dashed line). The red line marks the tangent to the droplet surface at the contact line. The white arc is the contact angle, θ, which exhibits partial wetting on glass (θ = 30 ± 10°), PEG-silane (θ = 25 ± 10°), and SLIPS (θ = 35 ± 10°) but complete dewetting on the PEGDA hydrogel (θ = 180 ± 3°). Substrates from left to right: bare glass, PEG-silane (3-[methoxy(polyethyleneoxy)propyl]trimethoxysilane), SLIPS of silicone oil (vinyl-terminated polydimethylsiloxane), and chemically cross-linked PEGDA hydrogel (molecular weight 12 kDa). Bottom: Schematics of the different surfaces. For SLIPS, we created a porous surface by coating the glass with hydrophobic nanoparticles (black points) and then infused this surface with a silicone oil (blue). The PEGDA hydrogel was covalently attached to the glass via a bonding layer (dark red) and formed by chemically cross-linking the highly hydrated PEG chains (green) in the presence of a photoinitiator and irradiating them with UV light (not shown). (B) Static contact angle measurements of various droplets in aqueous solution on the PEGDA hydrogel (top) versus PEG-silane (bottom). From left to right: side view of fluorescently labeled PDDA-STMP coacervate droplets, BSA-PEG droplets, DNA nanostar droplets, and a giant unilamellar vesile (GUV) in solution in the presence of a macromolecular depletant (PEG 100 kDa), resting on a PEGDA 12 kDa hydrogel (top, θ = 180 ± 3°) or PEG-silane bottom, (θ = 130 ± 5°). For PDDA and STMP droplets and BSA-PEG droplets on PEG-silane, 20% of the coated glass slides resulted in (θ = 180 ± 3°), whereas 80% of slides produced (θ = 130 ± 5°). All scale bars are 10 μm.

To evaluate the performance of the different coatings, we measured the static contact angle, θ, of protein droplets using 3D confocal fluorescence microscopy (Experimental Section).^35^ Contact angles 0 ≤ θ < 150° indicate wetting^36^ and hence significant interactions between the droplet and the surface. Complete dewetting occurs in the limit of θ = 180°, where the contact area and interactions between the droplet and surface are minimal. We evaluated the wetting performance of different surfaces using droplets of Laf1-AK-Laf1,^37,38^ which is a recombinant fusion protein of the DEAD-box protein Laf1 and adenylate kinase (AK), an enzyme involved in ATP energy transfer from mitochondria.^39^ The phase separation is driven by weak multivalent attractions between the intrinsically disordered regions (IDR) of Laf1,^40,41^ resulting in droplets with amphiphillic surfaces. We imaged the Laf1-AK-Laf1 droplets approximately 10 min after depositing them on the respective surface and found them to partially wet bare glass (θ = 30 ± 10°), PEG-silanized glass (θ = 25 ± 10°), and SLIPS (θ = 35 ± 10°) (Figure 1A). On bare glass, the contact angle continued to decrease beyond 10 min until complete wetting was achieved. In contrast, the PEGDA hydrogel exhibited complete dewetting of Laf1-AK-Laf1 droplets (θ = 180 ± 3°).

We then tested whether complete dewetting on PEGDA is a unique feature of Laf1-AK-Laf1 droplets by looking at the wetting behavior of five other phase-separating systems on the PEGDA hydrogel, using PEG-silanized glass as a control (Figure 1B). We selected the following systems, which spanned a wide range of interface chemistries and driving forces for phase separation.

#### Complex Coacervate

We used poly(diallyldimethylammonium chloride) (PDDA) and sodium trimetaphosphate (STMP). This combination of PDDA and a polyphosphate salt resembles the PDDA and ATP system, which has been used to study phase-separated microcompartments.^42^ Complex coacervates are driven by the electrostatic attraction of oppositely charged polyions and feature a highly hydrophilic surface.^43^

#### BSA-PEG Droplets

We selected the BSA-PEG system as a globular and segregative complement to the IDR-driven and associative Laf1-AK-Laf1 system. In this system, BSA and PEG of molecular weight 4 kDa undergo segregative phase separation in the presence of phosphate buffer (100 mM KH_2_PO_4_/K_2_HPO_4_, pH 7) and salt (200 mM KCl).^32^ Phase separation is driven by PEG-induced depletion interactions between BSA molecules, which themselves are negatively charged at pH 7, resulting in an amphipathic droplet surface.

#### DNA Nanostar Droplets

These DNA droplets assemble via specific interactions between complementary base pairs (hybridization) among so-called DNA nanostars.^44^ The resultant droplets feature a highly negatively charged surface due to the DNA’s phosphate backbone.

#### Giant Unilamellar Vesicles (GUVs)

We prepared GUVs in the presence of a depletion agent (PEG of molecular weight 100 kDa).^31,45^ GUVs are a model system for biological membranes (phospholipid bilayers) that has been widely used by the scientific community.^46^ Phospholipid bilayers are two-dimensional liquids that assemble from amphiphiles (e.g., palmitoyl-oleyl-phosphatidylcholine, POPC) and feature a hydrophilic surface that is determined by the lipid headgroups. The phosphatidyl choline headgroup of POPC is a zwitterion and carries no net charge.

#### Fluorinated Oil Droplets

We used FC-70 droplets in water, which have a highly hydrophobic surface. FC-70 was chosen for its relatively high surface tension in water (γ_FC-70_ = 60 ± 6 mN/m, as measured by pendant drop tensiometry), which is roughly 1000× higher than protein droplets and complex coacervates.^15,47^

In Figure 1B, we report the results of this analysis. Remarkably, we found that all systems showed negligible wetting or adhesion to the PEGDA hydrogel coating despite significant differences in their chemical properties. These results suggest that the PEGDA hydrogel is super-repellent to phase-separated liquids in aqueous buffers and provides an excellent substrate for studying protein, DNA, or complex coacervate droplets as well as phospholipid bilayers. The coating remains super-repellent in the presence of high-molecular-weight PEG (4 and 100 kDa). This suggests that PEG can readily diffuse into or weakly adsorb on the hydrogel because otherwise the depletion effect would induce adhesion of the droplets to the hydrogel surface.^48^ The PEGDA hydrogel is therefore uniquely suited for studies where PEG is used as macromolecular crowding agent.^31,45,49−51^ In comparison, droplets partially wetted PEG-silane in most cases (Figure 1B) with a contact angle of θ = 130 ± 5° for complex coacervates, BSA-PEG protein droplets, GUVs, and FC-70 droplets. PEG-silane performed well for DNA droplets (θ = 180 ± 3°). Occasionally, PEG-silanization yielded the complete dewetting of complex coacervates and BSA-PEG protein droplets. However, in our hands, this performance was achieved in only 20 of the 90 PEG-silanized glass slides we prepared (20%).

### Dynamic Contact Angle and Droplet Sliding

Sessile droplets exhibit a unique contact angle value when they have reached thermodynamic equilibrium on a defect-free and nonadapting surface.^52^ This is called the equilibrium contact angle. In most cases, however, the droplet is not in equilibrium due to contact line pinning, and the measured θ deviates from the equilibrium contact angle.^53^ Furthermore, the measured θ does not describe dynamics such as droplet sliding on a surface, which involves droplet wetting along its advancing edge and unpinning of the contact line at its receding edge. This is described by two contact angles, θ_*a*_ (advancing) and θ_*r*_ (receding), and their difference, *Δθ*, is known as contact angle hysteresis (Figure S7).^54,55^ Contact angle hysteresis is therefore more suitable for evaluating the mobility of a liquid droplet on a solid substrate. A greater hysteresis generally implies a decrease in droplet mobility and an increase in pinning and/or friction with the surface.

**Figure 2:**
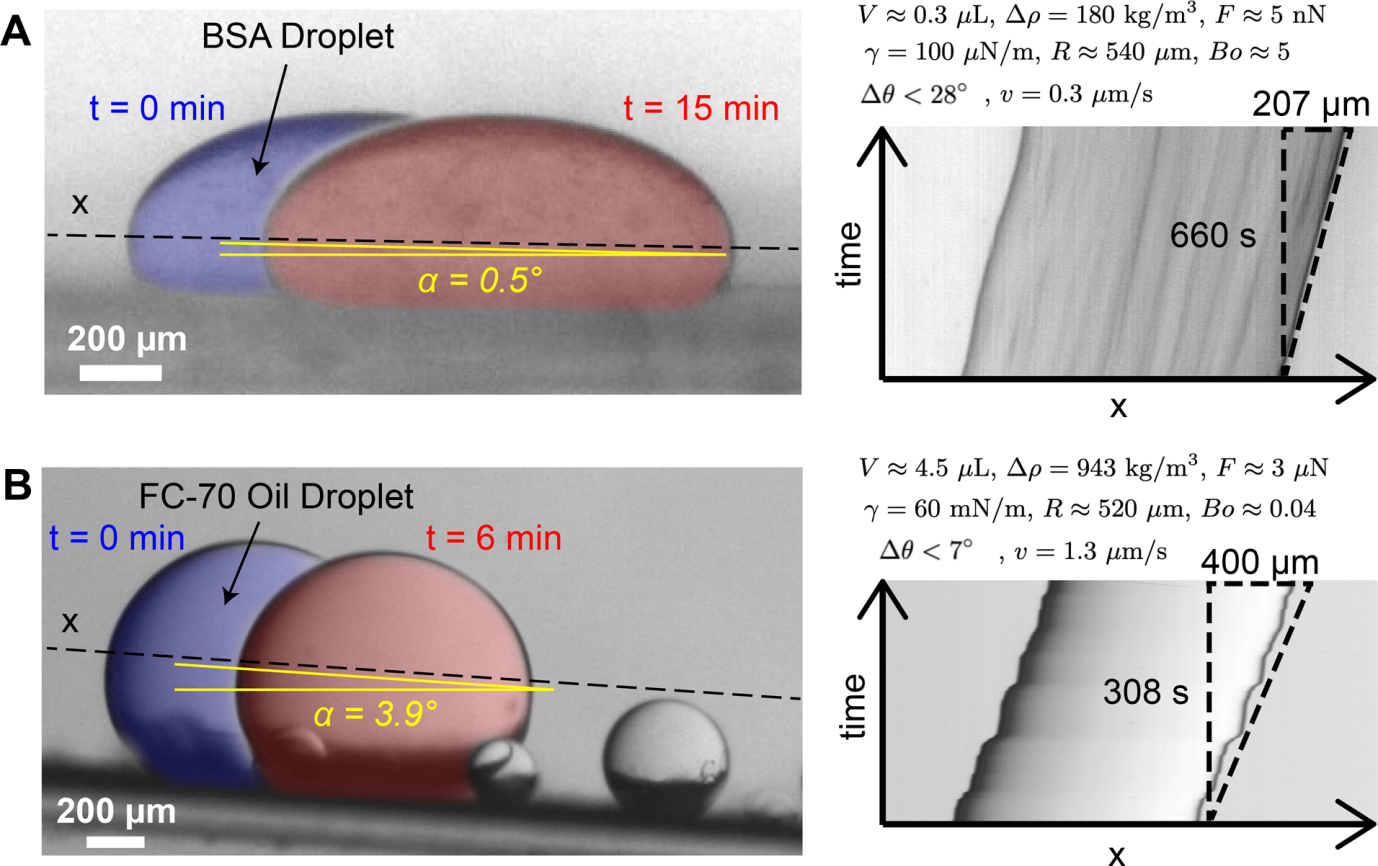
BSA and FC-70 droplets sliding on a PEGDA 12 kDa hydrogel-coated glass. (A) Left: Overlain images of a BSA droplet sliding on an inclined (α = 0.5°, yellow) PEGDA 12 kDa hydrogel at the beginning (*t* = 0 min, blue) and at the end (*t* = 15 min, red) of the time series. Right: Kymograph of the BSA droplet with velocity *v* = 0.3 μm/s. (B) Left: Overlain images of an FC-70 droplet sliding on an inclined (α = 3.9°) PEGDA 12 kDa hydrogel at the beginning (*t* = 0 min, blue) and at the end (*t* = 6 min, red) of the time series. Right: Kymograph of the FC-70 droplet with velocity *v* = 1.3 μm/s. The droplet volume has been estimated by considering droplets as ellipsoids that have a circular profile in the *x*–*y* plane. *V*, droplet volume; *Δρ*, density difference between a droplet and the surrounding solution; *F*, retention force; γ, interfacial tension between a droplet and the surrounding phase; *R*, droplet radius; *Bo*, Bond number; and *Δθ*, contact angle hysteresis. All values, except *Δρ* and γ, are obtained from the analysis of these images.

The two contact angles are typically measured by depositing a droplet on a flat horizontal substrate and then progressively tilting it.^55^ Initially, the droplet will deform under gravity but remain pinned in place by the retention force. At a critical value of tilting angle α, however, the droplet will start to slide down the substrate. The contact angles measured at the deformed advancing and receding ends of the droplet at the moment of motion onset are θ_*a*_ and θ_*r*_, respectively. A greater *Δθ* means greater droplet deformation and thus indicates stronger retention forces. First, we measured the critical tilting angles for BSA and FC-70 droplets in solution sliding on the PEGDA hydrogel (Figure 2). Inclinations of α < 1° were sufficient to make BSA droplets slide on the PEGDA hydrogel (Figure 2A), even for droplet volumes as low as ∼0.03 μL (Figure S5). Similarly, FC-70 droplets started to slide for α ≲ 4° on the hydrogel (Figure 2B). This suggests that minimal pinning occurs for both BSA and FC-70 droplets despite great differences in their chemical properties. To our knowledge, the critical tilting angles on the PEGDA hydrogel are smaller than those of the best superomniphobic and superoleophobic coatings reported in the literature, which generally require α values of a few degrees and droplet volumes of several microliters for sliding to occur.^56−59^

We can estimate the retention force, *F*, by equating it with the gravitational force component parallel to the surface at the onset of tilting, that is, at the critical tilting angle α
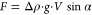
1where *Δρ* is the density difference between the droplet and the surrounding solution, *g* is the gravitational acceleration, and *V* is the droplet volume (Figure S7). Using eq 1 and *Δρ* = 180 kg/m^3^,^32^ we obtain an estimated retention force of *F* ≈ 5 nN for a typical BSA droplet of volume 0.3 μL (Figure 2A), which is at least 1 order of magnitude lower than the lowest values previously reported for droplets of similar size, including FC-70 droplets on SLIPS,^56^ water droplets on superhydrophobic surfaces,^60^ ethylene glycol droplets on silicon wafers,^61^ and water droplets on SOCAL.^62^ We found that BSA droplets with small volumes (e.g., 0.03 μL) could exhibit retention forces as low as 0.5 nN (Figure S5). For a typical FC-70 droplet of volume 4.5 μL, using *Δρ* = 943 kg/m^3^, we estimate *F* ≈ 3 μN (Figure 2B), which is comparable to the performance of SLIPS for various droplets in air (*V* ≈ 4.5 μL, *F* ≈ 1 μN).^56^

Indeed, our low retention forces are not directly comparable to the values reported for different liquids because the retention force is directly proportional to surface tension. Instead, droplet pinning is better quantified by *Δθ*, which is independent of surface tension and droplet size. However, it is challenging to accurately determine θ_*a*_ and θ_*r*_ by visual analysis of our relatively low resolution images because for such small values of α needed to trigger sliding, the droplets are essentially undeformed at the onset of motion.^60,63^ Under these circumstances, one can instead estimate the difference (cos θ_*r*_ – cos θ_*a*_) from the tilting angle α using a force balance between the retention force and gravity^59,64,65^ (details in Supporting Information)
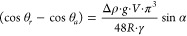
2where *R* is the droplet radius as seen from the side and γ is the interfacial tension of the droplet with respect to its surrounding phase (surface tension). Note that eq 2 assumes an elliptical contact line between a droplet and the substrate, which results from droplet motion.^65^

This relationship between contact angle hysteresis and the critical tilting angle is best captured by the Bond number, *Bo*, which quantifies the relative strength of gravitational forces which tend to move (and deform) the droplet to its surface tension:
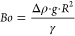
3Taylor expansion of cos θ around θ ≈ 180° and substituting  in eq 2 yields an upper bound for contact angle hysteresis:
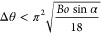
4For the BSA droplet in Figure 2A, eq 4 yields *Δθ* < 28° (using γ_BSA_ = 100 ± 30 μN/m)^32^ whereas for the BSA droplet in Figure S5 it yields *Δθ* < 11°. For the FC-70 droplet in Figure 2B, eq 4 yields *Δθ* < 7° (using γ_FC-70_ = 60 ± 6 mN/m). Since the above estimates are upper bounds, the true values for *Δθ* are expected to be significantly lower. As such, the PEGDA hydrogel performs well as a low-adhesion surface compared to SOCAL and SLIPS, for which 1° < *Δθ* < 10° values have been reported.^62^

**Figure 3:**
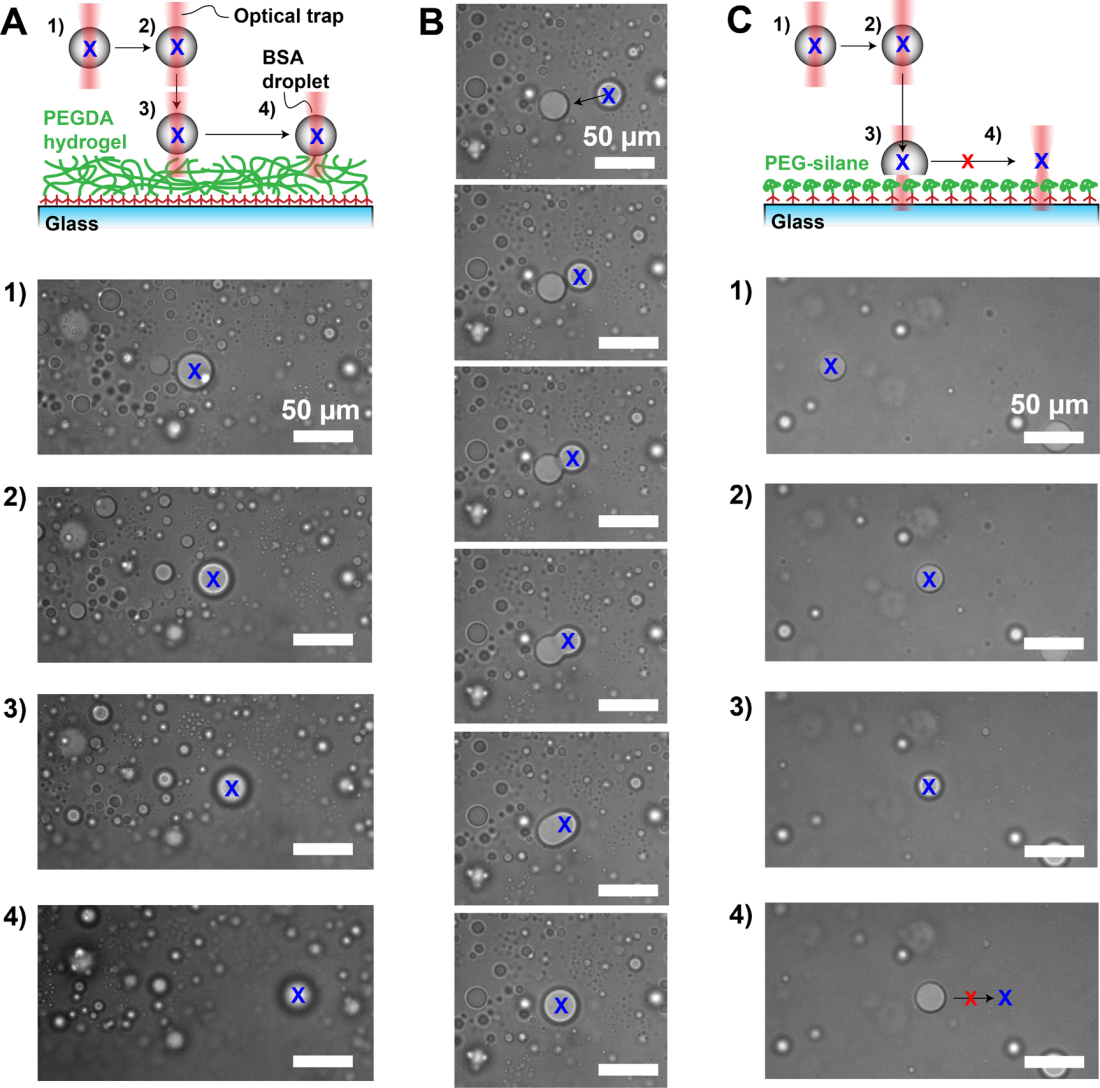
Image sequences for BSA droplet micromanipulation experiments with optical tweezers. (A) Top: Schematic of the micromanipulation of BSA droplets (gray) with an optical trap (red) on a PEGDA hydrogel (green). 1) We first grabbed a floating droplet from solution, 2) we then moved it to make sure the droplet was trapped, 3) the droplet was then lowered and gently pushed against the hydrogel, and 4) finally we moved the droplet on the hydrogel to verify the absence of pinning. Bottom: Bright field images corresponding to experimental sequence 1)–4). The location of the center of the trap is marked as a blue X. (B) Bright field image sequence of a trapped BSA droplet moved to another droplet and their subsequent fusion process. (C) Top: Schematic of the micromanipulation of BSA droplets (gray) with an optical trap (red) on PEG-silane (green). After trapping 1), moving 2), and lowering 3) the droplet, it partially wetted the PEG-silane, and we could no longer move droplet 4) (crossed out arrow). All scale bars are 50 μm.

We then tested whether the absence of pinning and/or friction extends to the smallest droplets. To this end, we used optical tweezers to manipulate microscopic BSA droplets of radius ∼10 μm (Figure 3). We trapped BSA droplets in solution and gently pushed them onto the coated glass slide surface and then tried to move the droplet along the surface. We were able to freely micromanipulate BSA droplets, even after they were pushed down onto the PEGDA hydrogel coating with the optical traps (Figure 3A), indicating that the dewetting behavior remains stable with respect to mechanical perturbations. In particular, we could bring droplets together to induce fusion (Figure 3B). The fusion process occurred freely for droplets resting on the PEGDA hydrogel. In contrast, BSA droplets brought down to a PEG-silane coating immediately wetted the surface and thus became immobilized (Figure 3C).

Taken together, the micromanipulation experiments, the small retention forces, and the static contact angles of nearly 180° convincingly demonstrate that protein and oil droplets on the PEGDA 12 kDa hydrogel behave similarly to water droplets on superhydrophobic surfaces under dynamic conditions.^59^ In other words, the interaction between droplets and the PEGDA hydrogel is minimal, and the droplets are free to roll and move with negligible pinning.

### Substrate Morphology and Stability

Hydrogels may experience surface adaption, in which their surface properties change over time.^66^ This occurs when the gel’s polymer chains undergo conformational changes to increase favorable interactions at the surface, for example, with a droplet resting on its surface.^52,67^ The appearance of such interactions would introduce adhesion and increase droplet wetting on the surface. In principle, such rearrangements could also occur at the surface of a droplet among its constituent macromolecules.

**Figure 4:**
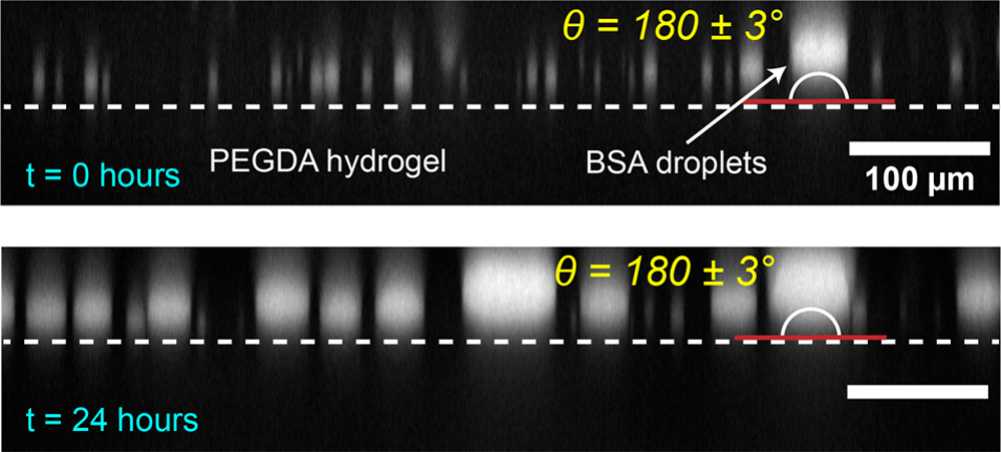
Coating stability and PEGDA hydrogel. Side view of BSA droplets resting on a PEGDA hydrogel over the course of 24 h at room temperature. Complete dewetting with a static contact angle of θ = 180 ± 1° is retained over the whole time period. The gel appears even and without defects over the entire field of view of approximately 1 mm^2^. Scale bars are 100 μm.

We tested the stability of the PEGDA hydrogel and whether surface adaption was taking place by recording static contact angles of BSA droplets over the course of 24 h and an area of 0.7 mm × 0.7 mm (Figure 4). We found that the BSA droplets remained dewetted on the PEGDA hydrogel for at least 24 h. These data show that the PEGDA hydrogel possesses sufficient stability for experiments lasting for prolonged time periods and that surface adaption is negligible on that time scale.

PEG-silane can be considered to be flat since it is only a few nanometers thick and thus follows the contour of the underlying glass slide.^68^ The SLIPS we use here has an intrinsic surface roughness stemming from variations in the depth of the nanoparticle matrix between approximately 30 and 500 nm or more.^69,70^ PEGDA hydrogels, on the other hand, are prepared by capillary spreading of the PEGDA-photoinitiator solution on a glass slide prior to curing, resulting in a thickness of several micrometers. They are also reported to undergo modest shrinkage of approximately 7% during curing,^71^ and in order to avoid wrinkling, the hydrogel layer had to be sufficiently thin. Moreover, we cured the hydrogel between two glass slides, one of which needed to be mechanically peeled off before use. The soft hydrogel would therefore be subjected to substantial stresses that could introduce defects, despite our use of an antistick coating. However, the hydrogel appears to be flat over the observed field of view (Figure 4). The hydrogel thickness is approximately 50 μm, which closely matches the expected value calculated from the glass slide dimensions (22 mm × 22 mm) and the pipetted volume of the PEGDA-photoinitiator solution (20 μL). Furthermore, the droplets appeared to sit on the surface of the gel and did not visibly penetrate it. Areas as large as 1 mm^2^ are easily found where the performances of the PEGDA gel are optimal and the morphology of the coating is relatively smooth and even.

During droplet sliding experiments, we found that over areas exceeding 1 mm^2^, PEGDA hydrogels can be uneven and have a few defects. While this is not particularly problematic for experiments with micrometer-sized droplets, as areas of the coating with good performance are typically larger than the field of view, it already becomes relevant for droplet sizes and trajectory lengths we observed for the dynamic contact angle measurements. The uneven kymograph accompanying the sliding FC-70 droplet in Figure 2B also suggests that the PEGDA hydrogel contains some unevenness or minor defects that weakly pin the oil droplet with separations of ∼100 μm. These defects were neither apparent in Figure 4 nor did they affect the sliding of the BSA droplets (Figure 2). However, in a few cases, we found visible defects in the form of crevices with depths of several micrometers, as shown in Figure S6, where the imperfections are visualized by the presence of numerous small droplets on the hydrogel surface. We attribute these imperfections to damage sustained by the gel during curing or by the excessive deformations induced in the hydrogel upon peeling of the top glass slide. We anticipate that the gel’s robustness can be significantly improved by optimizing the polymer content, amount of cross-linker, curing time, and gel thickness.

### Conditions for Dewetting

The remarkable consistency of dewetting across a variety of chemically distinct droplet systems and the lack of surface adaption over time strongly suggest that the mechanism is independent of the droplet’s detailed chemical properties. To rationalize this robust behavior, we adopt a simplified picture of a hydrogel as a porous material with a polymer volume fraction, ϕ. This allows us to use the Cassie–Baxter equation to express the apparent contact angle θ_*app*_ of a droplet resting on the hydrogel^72,73^
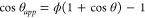
5where θ is the droplet’s contact angle with a surface comprising pure polymer (ϕ ≈ 1). Dewetting requires cos(θ_*app*_) → −1, which can readily be satisfied for θ > 90° and sufficiently small ϕ. For highly swollen hydrogels (ϕ ≪ 1), complete dewetting should occur when the polymer has even a slight preference for buffer over droplet constituents.

Equation 5 suggests that most swollen hydrogels will create a super-repellent surface for droplets in aqueous buffer. However, cross-linked polyethylene glycol (PEG) moieties are particularly suitable because they are neutrally charged and extremely hydrophilic. Instead of interacting with hydrophilic molecules (e.g., polar side chains) or sticking to hydrophobic molecules (e.g., fluorinated oil), PEG prefers to be hydrated by water. In addition, PEG does not engage in electrostatic interactions with charged polymers because it is neutral. As a result, PEG does not stick to most proteins, nucleic acids, complex coacervates, or lipid membranes.

## Conclusions

We developed a novel surface coating that repels droplets in aqueous solution while being optically transparent and thus ideally suited for light microscopy. Our coating is based on a chemically cross-linked PEGDA hydrogel that is covalently bound to a microscope glass slide. Complete dewetting on the PEGDA hydrogel occurred for droplets with remarkably diverse chemical properties including depletion-induced BSA droplets, IDR-containing protein droplets, complex coacervates, DNA nanostar droplets, fluorinated oil droplets, and phospholipid bilayers in the presence of a macromolecular crowding agent, PEG. All systems exhibited highly spherical shapes with static contact angles of θ = 180 ± 1°. In addition, we found minimal pinning or friction of BSA droplets and FC-70 droplets on the PEGDA hydrogel.

PEGDA hydrogels thereby offer a long-sought surface treatment with vanishing adhesion to a wide range of aqueous dispersions. Furthermore, the use of photomasks during curing allows the creation of complex geometries along with micropatterning of covalently attached ligands on the gel surface.^74^ Our method can be applied to commercially available glass-bottom sample chambers and 96-well plates. However, further development may be needed to achieve flat hydrogels in chambers where it is not straightforward to sandwich the PEGDA solution between a glass bottom and another surface during curing.

Future work should address the underlying mechanism of dewetting on these hydrogels by comparing different molecular weights of PEGDA based on their dewetting performance, degree of swelling, surface roughness, and mechanical modulus and whether other hydrogels can yield similar dewetting performance. More robust and scalable coatings of this kind could find applications as antifouling surfaces for biomedical and environmental applications.

## Experimental Section

### PEGDA 12 kDa Synthesis

PEGDA can be synthesized by starting from PEG at the desired molecular weight and acryloyl chloride.^75^ The objective of the synthesis is to attach an acrylate group from the acryloyl chloride molecule to each end of the poly(ethylene glycol) (Movie S1). While PEGDA can be easily found commercially at large molecular weights, it is 2 orders of magnitude more costly than synthesis from PEG and acryloyl chloride. Furthermore, when using commercially available PEGDA, an excess of photoinitiator must be added during the hydrogel curing step to consume any inhibitor contained in the formulation. A custom-made synthesis allows easier selection of the final molecular weight and yields a product that is more pure.

We started by weighing 10 g (0.83 mmol) of 12 kDa PEG (Alfa Aesar, 042635-30) and placing it in a Schlenk flask containing a magnetic stirrer. We removed oxygen and humidity from the flask by evacuating and purging it four times with nitrogen. Then all reactants were added under the protection of nitrogen. First, we injected 68 mL of anhydrous dichloromethane (DCM) (Acros Organics, 34846) into the flask to yield a final solution of 10% (w/w) PEG. Next, we injected 0.27 mL (3.32 mmol) of acryloyl chloride (Merck, 800826) dropwise into the flask, yielding a 2:1 molar ratio of acryloyl chloride to the hydroxy end-groups on the PEG. The reaction between PEGDA and acroyl chloride is reversible. We therefore injected 0.46 mL (3.32 mmol) of triethylamine (VWR, 28745.296) (in a 1:1 ratio with acroyl chloride) to quench the reverse reaction. We then covered the flask with aluminum foil and left it stirring overnight.

The next day, we transferred the contents of the Schlenk flask to a separatory funnel. A small amount of DCM was used to rinse the Schlenk flask to recover any residual PEGDA. Next, we added 1.5 M potassium bicarbonate to remove acidic byproducts and excess triethylamine. The volume of bicarbonate was between 1/4 and 1/2 of the total volume present in the funnel. Note that too much bicarbonate solution will lead to PEGDA dissolving in the aqueous phase, complicating its extraction. We shook the funnel vigorously while venting the funnel to release carbon dioxide between shaking. We then replaced the stopper of the funnel with parafilm and covered the body of the funnel with aluminum foil. The funnel was left vertical overnight to allow the phases to separate by gravity.

The next day, we drained the lower DCM phase, which contains most of the PEGDA, into a beaker. While stirring, we added anhydrous MgSO_4_ until the mixture went from a lumpy consistency to a well-dispersed mixture of powder and organic solvent. If too much MgSO_4_ is added, normal (not necessarily dry) DCM can be added to redissolve the PEGDA. We then prewetted two pieces of filter paper with DCM and placed them in a vacuum filter. The mixture was filtered through a Buchner funnel to remove MgSO_4_. The beaker was rinsed with DCM, and the wash was filtered to recover the maximum amount of PEGDA.

We then transferred the dried DCM phase into a round-bottomed flask and evaporated roughly half of the DCM with a rotovap (Rotovapor R-300, Bchi) at a temperature of 50 °C and a pressure of 690 mbar. Next, we prepared roughly 300 mL of cold diethyl ether in a beaker by placing the beaker in an ice bath. The solution from the rotovap was transferred to the cold diethyl ether in a dropwise manner using a pipet. A white solid immediately precipitated. The liquid was subsequently vacuum-filtered, and the powder remaining on the filter was further washed three times with cold diethyl ether. The powder was then transferred to a Petri dish and allowed to dry under a vacuum for ∼2 days.

### PEGDA 12 kDa Hydrogel Substrate Preparation

We pretreated the glass slides to allow chemical bonding of the PEGDA layer to the glass.^76^ We took 22 mm × 22 mm no. 1.5 thickness glass slides (VWR) and sequentially washed them once with Milli-Q water, ethanol, and Milli-Q water, followed by drying with an air gun. We then treated the glass slides with a UV-ozone cleaner (Bioforce Nanosciences, Pro-Cleaner) for at least 10 mintues in order to allow the formation of reactive OH bonds on the glass surface (Figure S4 (1)). In this step, UV-ozone treatment of the glass slides can be replaced by an alternative, such as plasma treatment or treatment with piranha solution. During UV-ozone cleaning, we prepared a solution of 900 μL of analytical grade ethanol (Fisher Chemicals) and 50 μL of Milli-Q water. We then added 3 μL of 3-(trimethoxysilyl) propyl methacrylate (Tokyo Chemical Industry, M0725) to the ethanol–water mixture, followed by brief vortex mixing. This compound is a silane coupling agent that consists of a silicon-containing moiety attached to an acrylate group. The silicon-containing end can react with OH groups on the glass surface to create a silicate bond and displace a water molecule, while the acrylate end can react with the cross-linker and be directly integrated into the PEGDA network. The solution was kept at room temperature on the bench for at least 5 min prior to use.

Within 5 min of removing the glass slides from the UV-ozone cleaner, we added 30 μL of the silane coupling agent to each slide. The solution was spread over the glass slides by moving each slide in different directions to ensure an even distribution of solution. The reaction was allowed to proceed for 3 min on the bench, after which it was quenched by placing the glass slides in a Petri dish containing analytical grade ethanol (Figure S4 (2)). The glass slides were then gently wiped with a Kimwipe wetted with analytical grade ethanol and subsequently dried with an air gun.

The next day, we prepared in a glass vial a 2 mL solution of photoinitiator 2-hydroxy-4-(2-hydroxyethoxy)-2-methylpropiophenone, also known as Irgacure 2959 (Merck, 410896), at a final concentration of approximately 2% (w/w). We wrapped the vial in aluminum foil to avoid exposure to light and sonicated it in a bath sonicator for 20 min at 55 °C to allow its complete dissolution. The solution was subsequently cooled to room temperature. During sonication, we prepared 600 μL of 12 kDa PEGDA stock at 30% (w/w) in Milli-Q water (Figure S4 (3)). We found that the smoothness of the final hydrogel increased with increasing PEGDA concentration, and we found that the solubility limit of 12 kDa PEGDA in water is about 40% PEGDA (w/w). We then mixed the initiator solution 1:1 with the 12 kDa PEGDA (600 μL each), with final concentrations of 1% (w/w) of photoinitiator and 15% (w/w) 12 kDa PEGDA.

We also prepared a second set of glass slides to cover the glass slides treated with PEGDA during UV curing. These new slides were, like before, washed with water, ethanol, and water and then dried with an air gun. We then rubbed these slides with a Kimwipe drenched in RainX Original Glass Water Repellent to create a nonstick coating and left these slides out to dry on the bench for a few minutes. For UV curing of the hydrogel, we added around 20 μL of the 1:1 PEGDA-photoinitiator solution to each pretreated glass slide (Figure S4 (4)) and then covered each one with a RainX-treated glass slide (Figure S4 (5)). In this way, a thin layer of the PEGDA-initiator mixture was allowed to spread via capillary action between the two glass slides, thereby creating a uniform coating. The resultant glass slide sandwiches were put under a UV lamp at wavelength λ = 365 nm and power 15 W (Analytik Jena, UVP XX 15BLB) for 1.5 h to allow complete curing of the PEGDA gel (Figure S4 (6)). The cured slides were stored in a closed Petri dish submerged in Milli-Q water until further use.

A glass sandwich was opened only immediately before experiment (Figure S4 (7)). Importantly, due to the permeability of the hydrogel to small solutes, it had to be equilibrated by soaking it for 30 min with supernatant stemming from the droplet suspension. After equilibration, the droplet suspension was added to the PEGDA hydrogel, and the system was sealed in order to avoid evaporation. To this end, we partially removed a portion of the PEGDA hydrogel using a razor to provide an adhesive surface for silicone isolators (Press-to-Seal, P24744) and then covered the sample with a cover glass. Under these conditions, the droplets and hydrogel could remain stable for prolonged time periods of ⩾24 h.

### PEG-Silane Substrate Preparation

First, we prepared the PEG-silanization solution by mixing 500 mL of toluene (Merck, 32249) and 2.3 mL of 2-[methoxy(polyethyleneoxy)propyl]trimethoxysilane, 90%, 6–9 PEG units (ABCR GmbH, AB111226) in a glass bottle followed by vigorous mixing. We then added 800 μL of aqueous HCl (37%) (VWR, 12463) to the solution. After being mixed thoroughly, the solution was carefully poured into a large crystallization dish placed under the fume hood while vigorously stirring with a magnetic stirrer.

Next, we washed microscope slides with water, ethanol, and water and then dried them with an air gun, as described in the PEGDA hydrogel procedure. As before, the clean glass slides were treated for at least 10 min with UV-ozone. The UV-ozone-treated glass slides were gently placed into the crystallization dish, making sure that the solution covers the coverslips and that the stir bar does not disturb the slides. If available, then a suitable glass rack may be used at this stage to immobilize and protect the glass slides inside the solution. The crystallization dish was covered with parafilm to prevent the evaporation of toluene and stirred in the fume hood at room temperature for 18 h. Afterward, the glass slides were rinsed once in toluene and then twice more with analytical grade ethanol before being dried with an air gun. Finally, the glass slides were stored in a dry chamber until required for use.

### SLIPS

Microscope slides were washed with water, ethanol, and water followed by drying with an air gun, as described above. Following a previously described procedure,^34^ we then sprayed silica nanoparticles (Glaco Mirror Coat Zero, Japan) onto the glass slides. The Glaco Mirror Coat comprises a suspension of hydrophobically modified silica nanoparticles in isopropyl alcohol, with a size distribution of 30–60 nm.^69,70^ After spraying the nanoparticles onto the glass slides, we waited 30 min to allow the alcohol to evaporate. The spraying and drying was repeated two more times, but for the last time, we let the coated glass slides dry for 24 h at room temperature, followed by 1 h under vacuum. The final step in preparing the SLIPS surface was to add a drop of generic silicone oil and to spin coat for 30 min at 1000 rpm. As silicone oil, we chose vinyl-terminated polydimethylsiloxane of viscosity 500 cSt (Gelest, DMS-V25), but we expect other variants such as methyl-terminated silicone oil to function equally well.

### Droplet Preparation

#### Production of Recombinant Laf1-AK-Laf1

Laf1-AK-Laf1 was purified as previously described.^38^ Shortly, the plasmid for recombinant expression was codon optimized for *E. coli*, synthesized, and cloned into a pET-15b vector by Genewiz (NJ, US). We fused *E. coli* adenylate kinase (AK) with the N-terminal LCD from Laf1 from *Caenorhabditis elegans* (AA 1-168). *E. coli* BL21-GOLD (DE3) cells were used for recombinant expression. Recombinant expression was induced at an OD of 0.7 with 0.5 mM isopropyl d-thiogalactopyranoside (99%, PanReac AppliChem) and grown for an additional 16 h at 37 °C. Cells were lysed by 15 × 60 s sonication pulses on ice with 120 s cooling breaks. Cells were lysed at pH 7.5 in 50 mM Tris-HCl, 10 mM imidazol (Sigma, Switzerland), 2 mM 2-β-mercaptoethanol (Sigma, Switzerland), and 500 mM NaCl (all reagents were obtained from Sigma, Switzerland). Laf1-AK-Laf1 was purified by immobilized metal ion affinity chromatography (Chelating Sepharose, GE Healthcare) according to a standard protocol. The protein was further purified by size exclusion chromatography using a Superdex 75 16/600 column (GE Healthcare) assembled on a KTA Prime system (GE Healthcare) using 50 mM Tris at pH 7.5 and 500 mM NaCl as an eluent buffer. The final purity of the proteins was assessed by SDS-PAGE electrophoresis. Protein stocks were concentrated to ∼1000 μM, and aliquots (20 μL) were frozen and stored at −20 °C until use. To initiate condensate formation, the stock solution was diluted to a final concentration of 5 μM in a Tris-HCl pH 8.0 buffer without salt. We formed droplets at 5 μM Laf1-AK1-Laf1 in 30 mM Tris-HCl (pH 8), 2.5 mM NaCl, and 5 mg/L rhodamine G, resulting in droplets enriched in Laf1-AK-Laf1 and rhodamine suspended in supernatant depleted of protein and dye.

#### Complex Coacervate

The coacervate droplets were formed by making a solution of 20 mM poly(diallyldimethylammonium chloride), 35% solution (Merck, 522376), 6.7 mM sodium trimetaphosphate (Merck, T5508), and 50 μM Alexa Fluor 647 (ThermoFisher, A20006) in Milli-Q water. The concentrations of the polycation and polyanion were chosen to yield a 1:1 molar ratio of positively and negatively charged moieties, respectively. The dye was added after the PDDA but before the STMP. This yielded droplets enriched in PDDA, STMP, and dye suspended in an aqueous solution depleted in these components.

#### BSA-PEG Droplets

BSA-PEG droplets were prepared as previously described.^32^ Briefly, we formed the droplets by preparing a solution of 100 mM potassium phosphate, pH 7, (Acros Organics, 42420 and Merck, 60349), 200 mM KCl (Merck, 60128), 30 g/L bovine serum albumin (BSA) (Merck, A7638), and 230 g/L PEG 4000 (Alfa Aesar, A16151) in Milli-Q water. In order to visualize the droplets with fluorescence imaging, a small fraction of the BSA was fluorescently labeled using Alexa Fluor594 NHS ester (ThermoFisher, A20004). This resulted in droplets enriched in BSA suspended in a supernatant enriched in PEG, both containing phosphate buffer and salt.

#### DNA Nanostar Droplets

The DNA droplets are composed of nanostars, each of which consists of four double-stranded DNA oligomers (Integrated DNA Technologies) that hybridize to form a cross shape. The nanostars have short, sticky palindromic overhangs on each arm, which provide weak specific attractions between nanostars with the same overhangs and thus drive droplet formation.

DNA nanostar droplets were prepared as previously described.^77^ Briefly, we first generated the DNA nanostars by mixing together the four oligomers, each at 50 μM in 10 mM Tris-HCl (pH 7.5), heated to 95 °C, and then annealed by cooling to 4 °C at −0.5 °C/s. We created two species of nanostars that do not bind to each other due to incompatible overhang sequences and a third species which is a mix of the two overhang sequences and can thus bind both species. To visualize the nanostars, we added a small fraction (1 mol %) of oligomers carrying a covalent fluorescent label, Cy3 and fluorescein (Integrated DNA Technologies) for the first and second species, respectively. We generated droplets by mixing together the nanostar stock solutions (1:1:0.5 molar ratio among the first, second, and third species, respectively) and diluting to 1 μM in 10 mM Tris-HCl (pH 7.5) and 500 mM NaCl. We then heated the samples to 50 °C for 30 min and cooled to room temperature. Imaging was performed after 1 h. This resulted in DNA nanostar droplets enriched with DNA nanostars suspended in a supernatant depleted in the nanostars, both containing the Tris buffer and salt.

#### Fluorinated Oil Droplets

We prepared 3 M Fluoriniert FC-70 droplets in 1 mL Milli-Q water by adding 5 μL of FC-70 and 1 μL of 1 mg/mL rhodamine G stock solution in ethanol. The solution was then vortex mixed, yielding a suspension of FC-70 droplets suspended in a solution of water and rhodamine G. A 20 μL portion of this suspension was immediately transferred into the imaging chamber. The confocal fluorescence images were inverted to better visualize the droplets since the fluorescent dye strongly partitioned into the aqueous phase.

#### GUV Preparation

We made the GUVs with the electroformation technique.^78,79^ We first prepared a 1 mM solution of lipids, with a lipid composition of 99 mol % 1-palmitoyl-2-oleoyl-*sn*-glycero-3-phosphocholine (POPC) (Avanti Polar Lipids, 850457C) and 1 mol % 1,2-dioleoyl-*sn*-glycero-3-phosphoethanolamine-*N*-(lissamine rhodamine B sulfonyl) (ammonium salt) (Rh-DOPE) (Avanti Polar Lipids, 810150C). 50 μL of this solution was deposited on an ITO plate using a glass syringe (Hamilton). A PDMS spacer was placed on the ITO plate, and a second ITO plate was placed on top. The chamber was filled with a solution of 280 mOsm/kg sucrose (BioXtra, ⩾99.5%) (Merck, S7903) and sealed.

The ITO plates are electrically connected to a signal generator (Keysight 33210A). The electroformation protocol consists of a gentle increase in AC voltage over the course of 25 min from 0 to 5 V at a fixed frequency of 10 Hz. After the voltage reaches 5 V, it is left for 2 h. Then, keeping the voltage at 5 V, the frequency is decreased to 5 Hz and left for another 30 min. The specific voltages, frequencies, and treatment times are listed in Table S1. The chamber was then disassembled, and the solution containing vesicles with varying sizes between a few μm and up to 50 μm was transferred to a glass vial and stored up to a few weeks. To prepare GUVs for fluorescence microscopy, 10 μL of the GUV solution was added to ∼100 μL of depletion medium, which consisted of 10 mM NaCl (VWR, 27810), 290 mOsm/kg d-(+)-glucose (BioXtra, ⩾99.5%) (Merck, G7528), and 0.68% (w/w) PEG 100 kDa (Merck, 181986), and then transferred to the sample chamber for imaging.

#### 3D Confocal Imaging and Contact Angle Measurements

We obtained *z* stacks of fluorescently labeled droplets resting on substrates (Figure 1) with a microscope (Nikon, Eclipse Ti2) equipped with a spinning disk confocal system (Yokagawa CSU-X1) using a 40× oil immersion objective lens (Nikon, MRH01401) with a 0.3 μm step size along the *z* axis. Images with a larger field of view (Figure 4) were acquired using a 20× air immersion objective lens (Nikon, MRH08230) with a step size of 2.5 μm. We processed the *z* stacks with ImageJ software to derive orthogonal views of the droplets.^80^ For the large field of view, we additionally performed a maximum intensity projection along one place in order to display more droplets in the figure. We corrected for optical aberrations along the *z* axis, which appeared due to the refractive index mismatch between the aqueous sample solution and the objective media.^81,82^ Specifically, we multiplied the nominal *z*-step size by the factor *k* = 0.85 for the immersion oil and *k* = 1.33 for air.

We used the angle tool in ImageJ to measure the contact angles of the corrected image. The measurement uncertainty was obtained by a visual inspection of the range of contact angles that was consistent with the image. In Figure S3, we show the six different droplet systems dewetting on the PEGDA hydrogel, with contact angles of θ = 180° and θ = 177° to guide the eye, resulting in an uncertainty of ±3° for all dewetted droplet systems.

### Sliding Contact Angle Measurements

The droplets were imaged using an optical setup with a white 3.5″ × 6″ LED backlight (Edmund Optics), a 0.5–1.0× VariMagTL Telecentric Lens (Edmund Optics), and a CMOS camera from Thorlabs (DCC3240M high-sensitivity USB 3.0, 1280 × 1024, global shutter, monochrome sensor). The use of a telecentric lens is important to ensure accurate size measurements. Samples were held in conventional polystyrene spectrophotometry cuvettes in which a piece of the coated glass was placed. The cuvette was filled with the continuous phase (supernatant), into which droplets of the coexisting dispersed phase were transferred using a micropipette. Tilting angles were adjusted by using a Thorlabs goniometer.

### Droplet Micromanipulation with Optical Tweezers

Experiments using optical tweezers were performed with Nikon Ti Eclipse inverted microscopes using a 60× water immersion objective lens. The trapping laser (Omicron Laserage Laserprodukte GmbH, LUXX 785-200 laser) had a wavelength of 785 nm and a maximum power of 200 mW. Images were captured with a Hamamatsu ORCA-Flash 4.0, C13440. The laser output was vertically polarized and sent through a half-wave plate and a polarizing beam splitter. The laser power at the sample and thus the trap strength were controlled by appropriately orienting the half-wave plate. After the beam splitter, the light was given circular polarization using a quarter-wave plate.

### Measurement of the Surface Tension of FC-70 in Water

We measured the interfacial tension of FC-70 droplets in water using a homemade pendant-droplet tensiometry setup. We suspended droplets from a G18 needle in a cuvette filled with Milli-Q water. We imaged the stable suspended droplets with the same optical setup as for sliding contact angle measurements and analyzed them with axisymmetric drop shape analysis.^83^ We used ρ_H_2_O_ = 997 kg/m^3^ and ρ_FC-70_ = 1940 kg/m^3^ (i.e., Δρ = 943 kg/m^3^).

## Supporting Information Available

Derivation of estimate for contact angle hysteresis from dynamic contact angle measurements, additional experimental details, and characterizations of surface morphologies and wetting (PDF)
Movie describing the process of PEGDA synthesis from acryloyl chloride and PEG (MOV)

## Supplementary Material

la3c01886_si_001.pdf

la3c01886_si_002.mov
