# Supplementary material for: Surface Passivation Method for the Super-repellence of Aqueous Macromolecular Condensates
Source: Langmuir. 2023 Oct 5;39(41):14626–37. doi: 10.1021/acs.langmuir.3c01886 (PMC10586374; doi:10.1021/acs.langmuir.3c01886)
Supplement: Supplementary file 1 — la3c01886_si_001.pdf [file la3c01886_si_001.pdf]

Supporting Information: Surface Passivation Method for Super-repellence of Aqueous Macromolecular Condensates

Andrea Testa,[†,||] Hendrik T. Spanke,[†,||] Etienne Jambon-Puillet,[†,‡] Mohammad Yasir,[†] Yanxia Feng,[†] Andreas M. Küffner,[¶] Paolo Arosio,[¶] Eric R. Dufresne,[†] Robert W. Style,[†] and Aleksander A. Rebane[*,†,§]

†*Department of Materials, ETH Zürich, 8093 Zürich, Switzerland.*

‡*LadHyX, CNRS, Ecole Polytechnique, Institut Polytechnique de Paris, Palaiseau 91120, France*

¶*Department of Chemistry and Applied Biosciences, Institute for Chemical and Bioengineering, ETH Zürich, 8093 Zürich, Switzerland.*

§*Life Molecules and Materials Lab, Programs in Chemistry and in Physics, New York University Abu Dhabi, P.O. Box 129188, Abu Dhabi, United Arab Emirates.*

||*These authors contributed equally.*

E-mail: a.rebane@nyu.edu

Derivation of estimate for contact angle hysteresis from dynamic contact angle measurements, additional experimental details, and characterizations of surface morphologies and wetting.

Dynamic contact angle

Contact angle hysteresis is the difference between the contact angle of a droplet sitting on a surface in wetting and dewetting regions. While on perfectly defect-free, ideal surfaces the contact angle θ is a unique value, on a real surface and in dynamic conditions, θ varies between two extremes, θ_a and θ_r, known as the advancing and receding contact angles, respectively[1,2] . If we imagine a droplet sitting on a flat surface, its shape will be symmetrical. Let's now tilt the surface at an angle α. There is a force, the *pinning force*, between the droplet and the surface, which will maintain the droplet adhered to the surface. Hence, it will initially not move, but it will start deforming under its own weight (Fig. S7). When a critical value of α is reached, however, the droplet will start sliding. Because of the deformation, the contact angles at the advancing and receding ends will be different: these are θ_a and θ_r. The stronger the pinning force, the more the droplet will deform before starting to move, hence the larger the difference $\Delta\theta = \theta_a - \theta_r$.

The values of θ_a and θ_r can be calculated by a simple force balance[3] . A side-view of the droplet is represented in Fig. S7. The droplet slides as a result of the parallel component of the gravitational force with respect to the surface, $F_{G\parallel}$, which be expressed as

$$F_{G\parallel} = \Delta\rho \cdot V \cdot g \cdot \sin\alpha, \quad (1)$$

where $\Delta\rho$ is the density difference between the droplet and the surrounding medium, V the droplet volume, g the gravitational acceleration, and α the tilting angle.

The pinning force F, on the other hand, counteracts droplet motion. This should be derived in principle from the droplet surface tension balance along the whole droplet contact line. In most studies, however, its value is simply determined by the balance of forces at the advancing (f_a) and receding (f_r) ends, multiplied by a geometrical pre-factor, k:

$$F = k \cdot (f_r - f_a). \quad (2)$$

Several values of k have been reported in literature[4] . For droplets with circular contact area, $k = \frac{48}{\pi^3} R$ offers a good approximation, where R is the droplet radius as seen from the side[5] . By expressing f_a and f_r in terms of the droplet surface tension γ, we can then rewrite Eq. 2 as

$$F = \frac{48R}{\pi^3} \cdot (\gamma \cos\theta_r - \gamma \cos\theta_a). \tag{3}$$

At the onset of motion, $F_{G\|} = F$, hence:

$$\Delta\rho \cdot g \cdot V \cdot \sin\alpha = \frac{48R}{\pi^3} \cdot \gamma \cdot (\cos\theta_r - \cos\theta_a), \tag{4}$$

which then can be rearranged as

$$(\cos\theta_r - \cos\theta_a) = \frac{\Delta\rho \cdot g \cdot V \cdot \pi^3}{48R \cdot \gamma} \cdot \sin\alpha. \tag{5}$$

Importantly, we can estimate the retention force using Eq. 1 by measuring the critical tilting angle α, the droplet volume V, the density difference $\Delta\rho$, and knowing the gravitational acceleration g. First, we substitute $V \approx \frac{4\pi R^3}{3}$ into Eq. 5:

$$(\cos\theta_r - \cos\theta_a) = \frac{\Delta\rho \cdot g \cdot R^2 \cdot \pi^4}{36 \cdot \gamma} \cdot \sin\alpha. \tag{6}$$

We note that the relevant dimensionless quantity is the Bond (or Eötvös) number:

$$Bo = \frac{\Delta\rho \cdot g \cdot R^2}{\gamma}. \tag{7}$$

Thus,

$$(\cos\theta_r - \cos\theta_a) \approx \frac{\pi^4}{36} \cdot Bo \cdot \sin\alpha, \tag{8}$$

that is, the critical tilt angles can only be used as a relative measure of contact angle hysteresis between droplets with similar Bond numbers. We then use the Taylor expansion

of $\cos\theta$ around $\theta \approx 180°$, to rewrite the left-hand side of Eq. 8 as follows:

$$(\cos\theta_r - \cos\theta_a) \approx \frac{1}{2}\left(\theta_r^2 - \theta_a^2 + 2\pi(\theta_a - \theta_b)\right). \tag{9}$$

The substitution $\theta_r = \theta_a - \Delta\theta$ yields

$$(\cos\theta_r - \cos\theta_a) \approx \frac{1}{2}\left(\theta_a^2 - 2\theta_a\Delta\theta + \Delta\theta^2 - \theta_a^2 + 2\pi\Delta\theta\right). \tag{10}$$

or

$$(\cos\theta_r - \cos\theta_a) \approx \frac{1}{2}\left(\Delta\theta^2 + 2(\pi - \theta_a)\Delta\theta\right). \tag{11}$$

Substituting Eq. 11 into Eq. 8 yields a quadratic equation for $\Delta\theta$:

$$\Delta\theta^2 + 2(\pi - \theta_a)\Delta\theta = \frac{\pi^4}{18} \cdot Bo \cdot \sin\alpha. \tag{12}$$

The positive solution for Eq. 12 is

$$\Delta\theta = (\theta_a - \pi) + \sqrt{(\pi - \theta_a)^2 + \frac{\pi^4}{18} \cdot Bo \cdot \sin\alpha}. \tag{13}$$

Taking the upper bound of the right-hand side of Eq. 13 yields

$$\Delta\theta < \pi^2\sqrt{\frac{Bo \cdot \sin\alpha}{18}}. \tag{14}$$

Supporting Figures

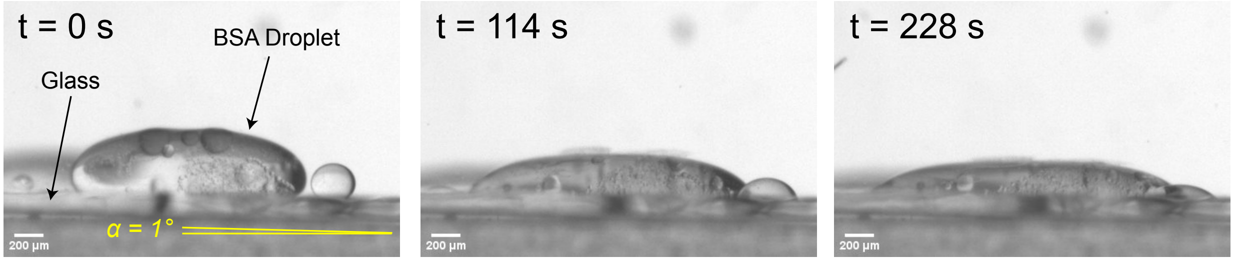

Figure S1: *Wetting and spreading of a BSA droplet on bare glass.*

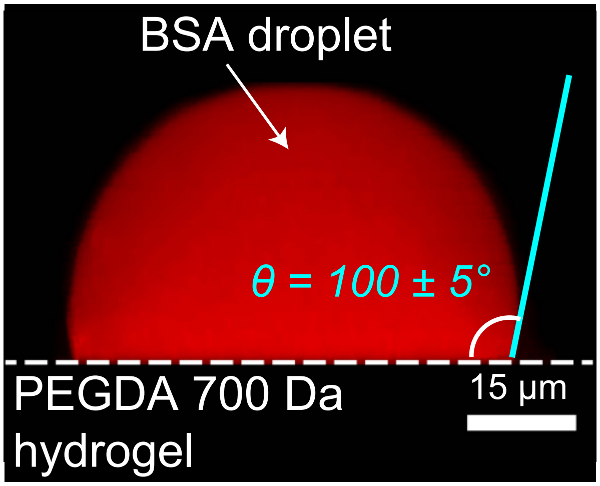

Figure S2: *Partial wetting of a BSA droplet on PEGDA 700 Da hydrogel with contact angle* $\theta = 100 \pm 5°$. Adapted from Testa et al.[6]

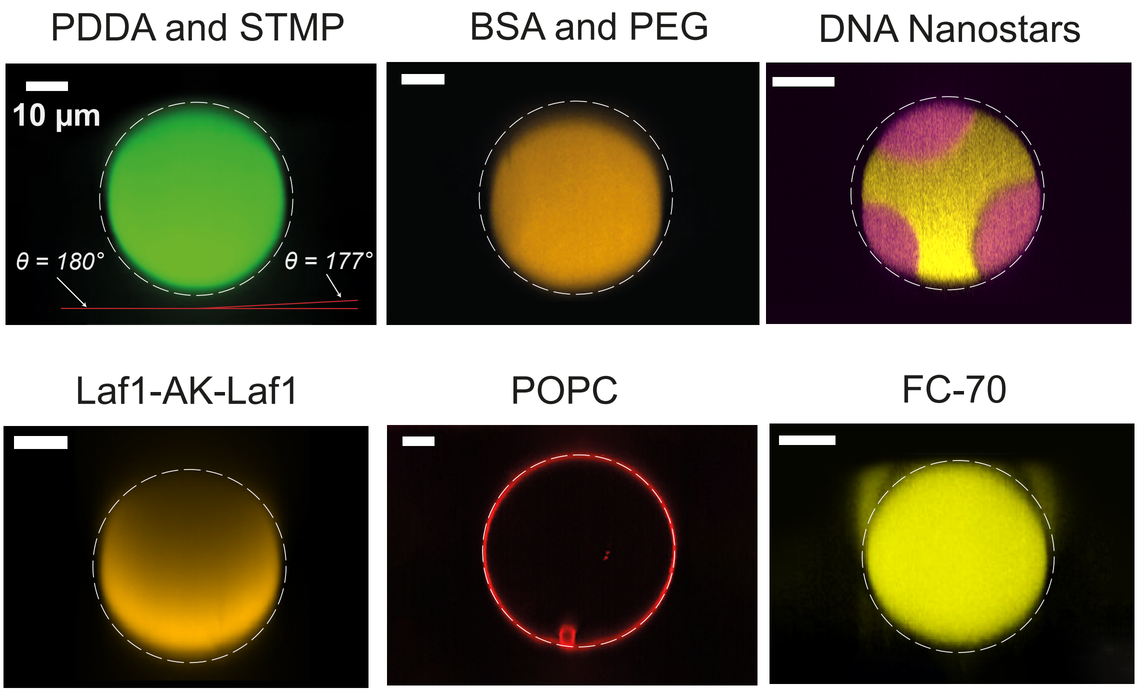

Figure S3: *Uncertainty in static contact angle for dewetted droplets.* The six different systems from Fig. 1 are shown with contact angles $\theta = 180°$ and $\theta = 177°$ drawn to show the uncertainty interval in measurement. The white dashed curve delineates a circle to emphasize that the drop remains spherical even at the point of contact at the bottom. All scale bars are 10 μm.

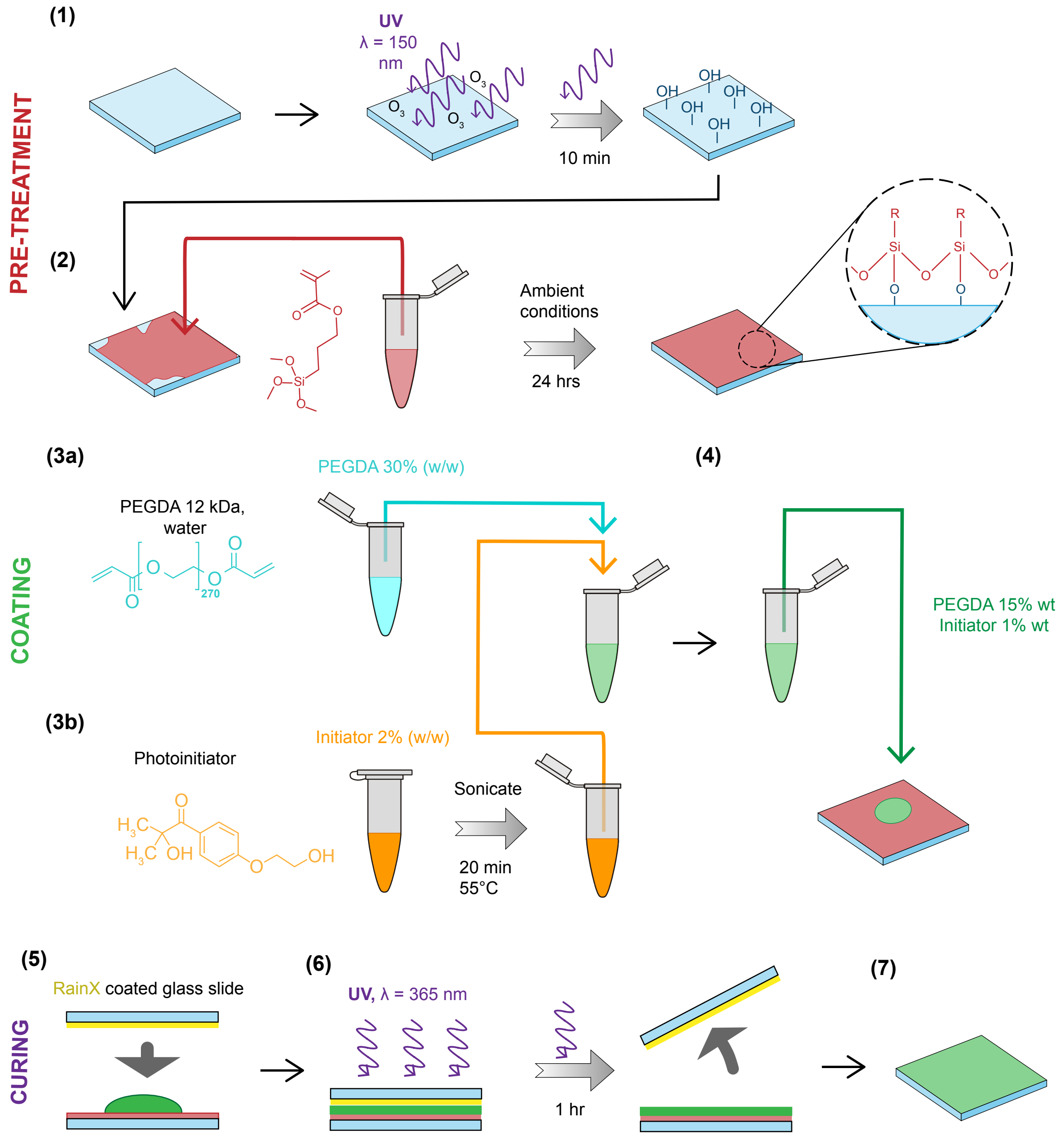

Figure S4: *PEGDA* 12 kDa *substrate preparation* **Pre-treatment (1)** Clean glass slides are treated with UV-Ozone for 10 minutes and then **(2)** coated with a solution containing 3-(trimethoxysilyl) propylmethacrylate (silane coupling agent, *red*). The reaction is allowed to complete over the course of 24 hours. **Coating (3a)** A solution of PEGDA 30 % by weight in water is made (*cyan*). **(3b)** At the same time, a solution of photoinitiator 2 % by weight in water is made (*orange*) and sonicated for 20 minutes at $55°C$ to assist solubilization. The PEGDA and photoinitiator solutions are then mixed together at 1:1 volume ratio (*green*) to yield final concentrations of 15% w/w PEGDA and 1% w/w photoinitiator and then **(4)** added to a pre-treated glass slide. **Curing (5)** The drop of the mixture is sandwiched between the pre-treated glass slide and a glass slide coated with RainX® (*yellow*) and then **(6)** cured under UV light for 1 hour. **(7)** The top glass slide is then removed to expose the cured PEGDA hydrogel.

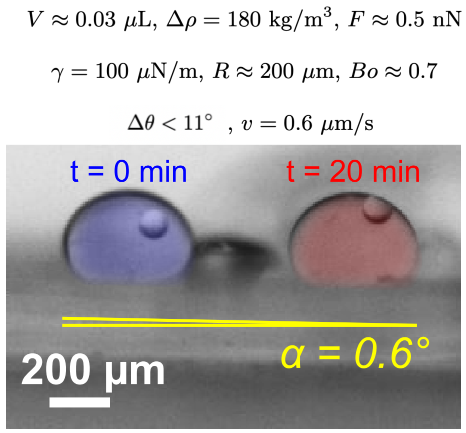

Figure S5: *Low critical tilting angle for BSA droplet sliding on PEGDA hydrogel.* BSA droplet of a small volume ($V \approx 0.03$ μL) with Bond number $B \approx 0.7$ sliding at very low tilting angle ($\alpha = 0.6°$) and velocity ($v = 0.6$ μm/s). The corresponding retention force is $F \approx 0.5$ nN and contact angle hysteresis is $\Delta\theta < 11°$.

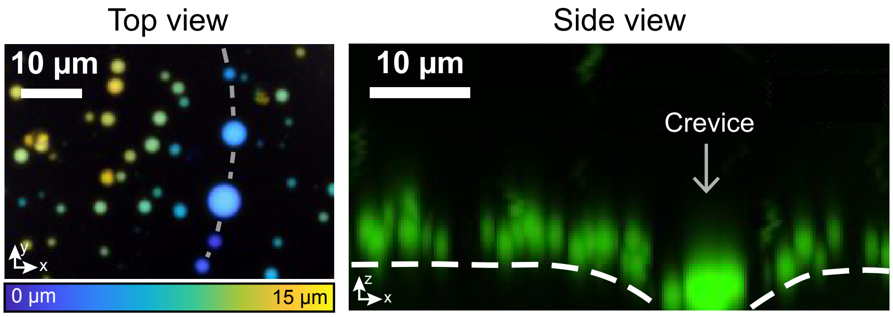

Figure S6: *Crevice-like defects on the surface of a PEGDA hydrogel.* Left side: z-projections of top view of an area of the hydrogel, color coded in function of the height from the lowest recorded z-plane. Droplets sitting lower appear blue, while droplets sitting higher appear green and then yellow. The dashed line represents the bottom of the crevice. Right side: side view of the same area of the sample. The dashed line represents the surface of the PEGDA hydrogel. All scale bars are 10 μm.

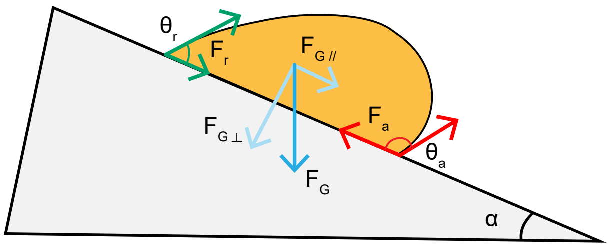

Figure S7: *Force balance of a droplet sliding on a tilted surface.* With advancing contact angle θ_a and receding contact angle θ_r

Table S1: AC treatment program for electroformation of GUVs

Step	Frequency	Voltage (peak-to-peak)	Time
1	10 Hz	830 mV	5 min
2	10 Hz	1.66 V	5 min
3	10 Hz	2.5 V	5 min
4	10 Hz	3.3 V	5 min
5	10 Hz	4.1 V	5 min
6	10 Hz	5 V	2 hours
7	5 Hz	5 V	30 min

Movie S1: Movie describing the process of PEGDA synthesis from acryloyl chloride and PEG.

References

(1) Shi, Z.; Zhang, Y.; Liu, M.; Hanaor, D. A.; Gan, Y. Dynamic contact angle hysteresis in liquid bridges. *Colloids and Surfaces A: Physicochemical and Engineering Aspects* **2018**, *555*, 365–371.

(2) Eral, H.; t Mannetje, D.; Oh, J. M. Contact angle hysteresis: a review of fundamentals and applications. *Colloid and polymer science* **2013**, *291*, 247–260.

(3) De Gennes, P.-G.; Brochard-Wyart, F.; Quéré, D., et al. *Capillarity and wetting phenomena: drops, bubbles, pearls, waves*; Springer, 2004; Vol. 315.

(4) Wong, T.-S.; Kang, S. H.; Tang, S. K.; Smythe, E. J.; Hatton, B. D.; Grinthal, A.; Aizenberg, J. Bioinspired self-repairing slippery surfaces with pressure-stable omniphobicity. *Nature* **2011**, *477*, 443–447.

(5) ElSherbini, A.; Jacobi, A. Retention forces and contact angles for critical liquid drops on non-horizontal surfaces. *Journal of colloid and interface science* **2006**, *299*, 841–849.

(6) Testa, A.; Dindo, M.; Rebane, A. A.; Nasouri, B.; Style, R. W.; Golestanian, R.; Dufresne, E. R.; Laurino, P. Sustained enzymatic activity and flow in crowded protein droplets. *Nature Communications* **2021**, *12*, 6293.
